# Impact of Androgen Suppression Therapy on the Risk and Prognosis of Bladder Cancer: A Systematic Review and Meta-Analysis

**DOI:** 10.3389/fonc.2021.784627

**Published:** 2021-12-14

**Authors:** Peng Xiang, Zhen Du, Yongxiu Hao, Di Guan, Dan Liu, Wei Yan, Mingdong Wang, Yutong Liu, Hao Ping

**Affiliations:** ^1^ Department of Urology, Beijing Tongren Hospital, Capital Medical University, Beijing, China; ^2^ Beijing Advanced Innovation Center for Big Data-Based Precision Medicine, Beihang University & Capital Medical University, Beijing Tongren Hospital, Beijing, China; ^3^ Department of Epidemiology and Biostatistics, School of Public Health, Peking University, Beijing, China

**Keywords:** androgen suppression therapy, bladder cancer, incidence, recurrence, meta-analysis

## Abstract

**Purpose:**

The purpose of this study was to summarize the existing evidence and develop a comprehensive systematic review of the impact of androgen suppression therapy (AST) on the incidence or clinical outcomes of bladder cancer.

**Methods:**

We systematically searched the PubMed and Embase databases from inception to June 20, 2021 to identify all observational studies examining the incidence or clinical outcomes of bladder cancer in patients who received AST. AST is defined as the use of 5-alpha reductase inhibitors (5-ARIs) or androgen deprivation therapy (ADT).

**Results:**

A total of 18 observational studies were included. Our results showed that AST was not significantly associated with a reduced risk of BCa incidence (OR: 0.92, 95% CI: 0.68–1.24) compared with the lack of AST. The subgroup analysis revealed that finasteride use was significantly associated with a reduction in the risk of BCa incidence (OR: 0.75, 95% CI: 0.64–0.88). Recurrence-free survival (RFS) was improved among AST users compared with nonusers (HR: 0.68, 95% CI: 0.48–0.95), while no significant difference between AST users versus nonusers was identified for cancer-specific survival (CSS), overall survival (OS) or progression-free survival (PFS).

**Conclusion:**

Current evidence indicates that therapy with finasteride may represent a potential strategy aimed at reducing BCa incidence. Moreover, AST has a beneficial effect on the recurrence of bladder cancer. Further well-designed randomized trials or cohort studies with better characterized study populations are needed to validate our preliminary findings.

**Systematic Review Registration:**

International Prospective Register of Systematic Reviews database [https://www.crd.york.ac.uk/PROSPERO/], identifier CRD42021261685.

## Introduction

Bladder cancer (BCa), predominantly urothelial carcinoma, is a common malignant genitourinary tumor (1, 2). Men are 3 to 4 times more frequently diagnosed with bladder cancer than women; however, women tend to be diagnosed with more advanced disease at presentation and have less favorable outcomes after treatment (1, 3–5). Female patients with urothelial carcinoma of the bladder have been shown to have worse cancer specific survival, overall survival and recurrence-free survival (5, 6). Recent studies question why there are differences, and the effects of sex hormones and its receptors, especially androgens, have become widely researched (1, 5–7).

Sex hormones and corresponding receptors are relevant modulators of cancer onset and progression in nonreproductive organs, particularly the lung, colorectal, bladder, stomach, kidney, pancreas, and thyroid gland (8). The excessive or reduced expression of these receptors, and the changes in their upstream or downstream pathways are closely related to the outcomes of BCa (8, 9). Numerous studies have focused on the role of androgen receptor (AR) and androgens in the development of bladder cancer. *In vitro* and *vivo* evidence highlights a crucial role for AR in BCa development, progression, recurrence and resistance to standard therapies such as chemotherapy, radiotherapy, and Bacillus Calmette Guerin (BCG) (2, 4, 8, 10–14). Emerging clinical evidence also suggests that the manipulation of androgen signaling may affect BCa behavior. Previous meta-analyses included limited clinical literature and some unreported relative risks in studies, suggesting that androgen suppression therapy (AST) consisting of 5-alpha reductase inhibitors (5-ARIs) or androgen deprivation therapy (ADT) can reduce BCa incidence, recurrence and specific mortality (15, 16). However, various results regarding the impact of AST on the incidence and recurrence of bladder cancer have been widely reported recently, and there are disputes among them. Therefore, with the increase in original research on this topic, an updated summary needs to be presented.

Herein, the aim of our study is to summarize the available evidence and develop a comprehensive systematic review of the effect of AST on the incidence of bladder cancer and the clinical outcomes of patients with bladder cancer.

## Methods

The protocol of this study has been registered in the International Prospective Register of Systematic Reviews database (CRD42021261685).

### Search Strategy and Eligibility Criteria

The PubMed and Embase databases were searched from inception to June 20, 2021. The following search terms were used: “bladder cancer,” “urothelial carcinoma,” or “bladder neoplasms”; one of “androgen suppression therapy” or “5 alpha reductase inhibitor” or “5α-reductase” or “5ARI” or “finasteride” or “dutasteride “ or “androgen deprivation therapy” or “anti-androgen” or “bicalutamide” or “enzalutamide” or “abiraterone” or “GnRH agonist” or “GnRH antagonist” or “castration” or “nilutamide” or “flutamide” or “apalutamide” or “darolutamide”. The titles and abstracts of articles were screened initially to identify relevant studies. Then, the full texts of potentially relevant studies were carefully read to determine those that met the eligibility criteria. Retrospective and prospective studies evaluating the effect of AST (5-ARI or ADT) on BCa incidence, recurrence, or survival were included in the analysis. Articles that did not report AST in patients with BCa were excluded. Reviews, letters, editorials, replies from authors, case reports, conferences and articles not published in English were excluded. Two authors screened the search results and any disagreements were resolved.

### Data Extraction and Quality Assessment

Data extracted from the eligible studies included study characteristics (e.g., study type, data source, study period, sample size,median of follow-up), patient characteristics (e.g., patient age, AST type), outcomes (e.g., BCa incidence, BCa recurrence), adjusted risk estimates with 95% confidence interval (CI) for outcomes, and potentially confounding factor adjustments (e.g., age, race, smoking, comorbidities tumor stage and grade, intravesical therapy). The main outcomes were ① incidence of BCa when AST was initiated before diagnosing BCa and ② recurrence-free survival (RFS), progression-free survival (PFS), overall survival (OS) or cancer-specific survival (CSS) when AST was initiated after diagnosing BCa. We used the Risk of Bias in Nonrandomized Studies of Interventions (ROBINS-I) tool to assess methodological quality and summarized the results in **Supplementary Table 1**.

### Statistical Analysis

The meta-analysis was performed by the Review Manager Version 5.3 software. Due to the observational nature of the included studies, we extracted adjusted hazard ratios (HRs) and odds ratios (ORs) with 95% CIs from the multivariate logistic regression analysis to calculate the cumulative effect size (17). Moreover, HRs and incidence density ratios can be regarded as relative risks (RRs) directly (18, 19). Additionally, ORs are close to RRs because of the low incidence of outcome (<10%) (20). The Cochrane Q test and *I^2^* were used to determine the level of heterogeneity among studies. In the case of heterogeneity (*p* < 0.10 or *I^2^* > 50%), the random effects model was used; otherwise, a fixed effects model was used. A *p* value < 0.05 was considered statistically significant. In addition, according to the type of AST, we performed a subgroup analysis of the effect of AST on BC incidence. Finally, publication bias was assessed by using a funnel plot when there were more than 10 studies that reported a specific outcome.

## Results

### Characteristics of Included Studies and Patients

**Figure 1:**
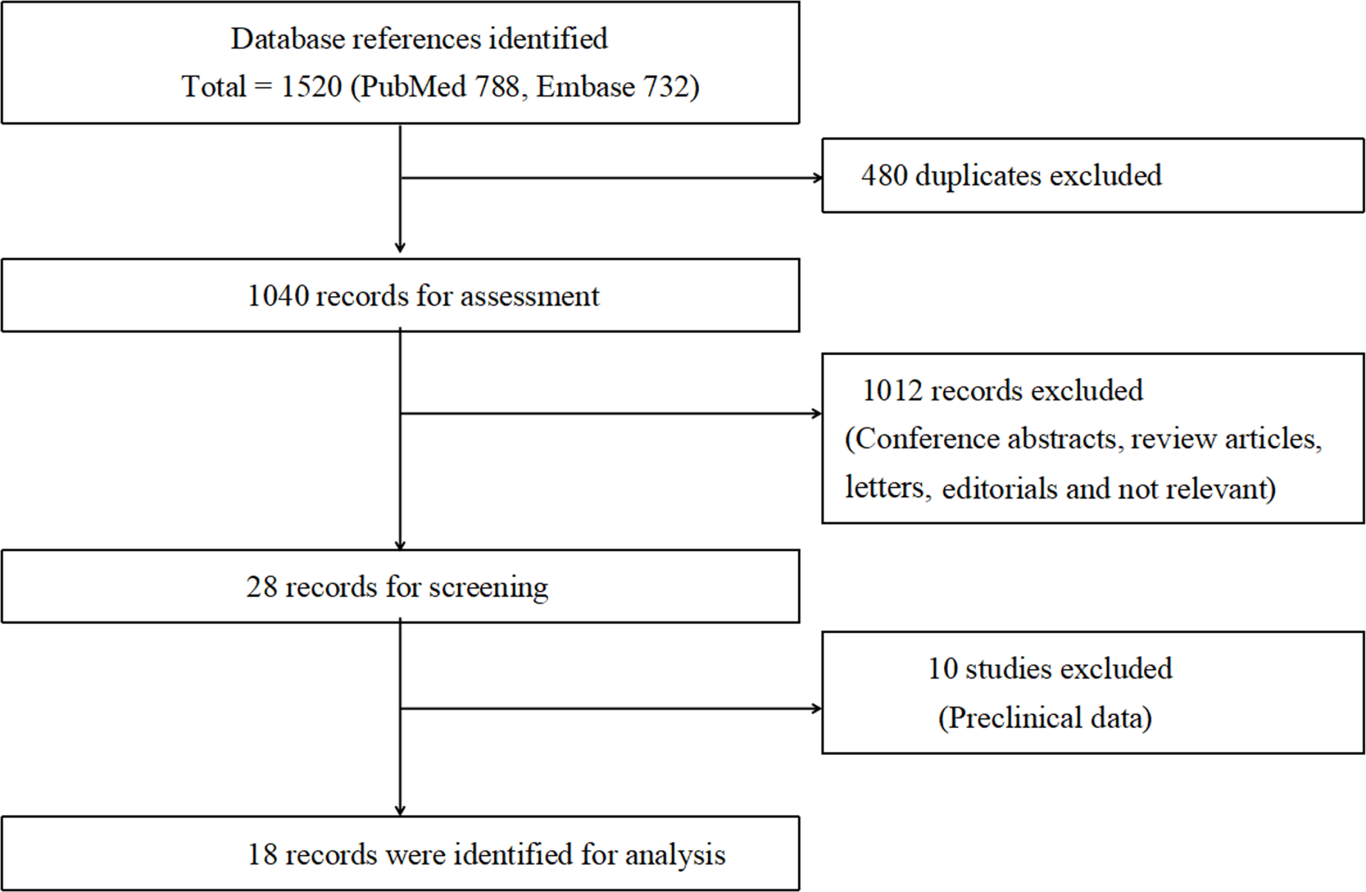
Flow diagram of the study.

Overall, according to the screening criteria, the systematic review and meta-analysis included 18 studies with a total of 414 007 male patients (21–38) (**Figure 1**). Eight studies evaluated the effect of AST on bladder cancer incidence (22, 27, 28, 30, 32–34, 38). Ten studies examined the effect of AST on bladder cancer recurrence, progression and survival (21, 23–26, 29, 31, 35–37). The characteristics of the selected studies were summarized in **Table 1**. The search strategy was presented in **Supplementary Table 2**. 

**Table 1:** Characteristics of the included studies.

Study, year, country	Study type	Date source	Study period	Sample size	Comparisons	Participants	Age (yr), mean	Key inclusion criteria	Follow-up (yr)	Outcome measures
Al-Hogbani, 2020, Canada (21)	Retrospective cohort	Chart review	2013-2018	206	5-ARIs No 5-ARIs	39 167	74 68	NMIBC treated with BCG	3.3	Bladder cancer recurrence and progression-free survival
Chen, 2018, Taiwan (22)	Case control	Administrative database	2002-2013	33586	Bladder cancer No bladder cancer	16784 16784	68.6 ± 13.0 68.6 ± 13.0	Diagnosis of patients with bladder cancer or without bladder cancer	6	Effect of 5-ARIs on bladder cancer incidence
Izumi, 2014, Japan (23)	Retrospective cohort	Chart review	1991-2013	162	ADT No ADT	86 76	74 (59-88) 71.5 (54-92)	Diagnosis of bladder and prostate cancer	5.2	Bladder cancer recurrence
Kufukihara, 2021, Japan (24)	Retrospective cohort	Chart review	1999-2017	48	ADT No ADT	29 19	NA NA	Diagnosis of NMIBC and prostate cancer	5	Bladder tumor recurrence
Mäkelä, 2018, Finland (25)	Retrospective cohort	Administrative database	1997-2012	10702	5-ARIs No 5-ARIs	1328 5090	78 (72-83) 70 (61-78)	Diagnosis of bladder cancer	4.2	Bladder cancer specific survival; The risk of multiple TURB procedures
McMartin, 2019, Canada (26)	Retrospective cohort	Chart review	2009-2017	338	5-ARIs No 5-ARIs	48 290	72.5 68.7	Patients with urothelial carcinoma undergo radical cystectomy	1.8	Bladder cancer survival,such as OS, CSS and RFS; Pathological features assessment including LVI and PNI
Morales, 2016, America (27)	Retrospective cohort	Trial database	1993-2001	72370	5-ARIs No 5-ARIs	6069 66 301	63 (55-78) 62 (49-78)	PLCO screening trial participants	13	Incidence of bladder cancer
Moschini, 2019, America (28)	Retrospective cohort	Administrative database	2000-2009	196914	ADT No ADT	68421 128493	75 (70-79) 71 (68-76)	Diagnosis of localized prostate cancer	4.9	Incidence of bladder cancer
Pastore, 2019, Italy (29)	Retrospective cohort	Chart review	2015-2017	312	5-ARIs No 5-ARIs	165 147	75.2 ± 10.5 75.1 ± 9.3	Diagnosis of NMIBC	2.5	Bladder tumor recurrence and survival
Sathianathen, 2018, America (30)	Retrospective cohort	Trial database	1992-1998	2700	5-ARIs No 5-ARIs	1216 1484	62.6 ± 7.2 62.6 ± 7.4	MTOPS LUTS study participants	6	Incidence of bladder cancer
Shiota, 2017, Japan (31)	Retrospective cohort	Chart review	2010-2013	228	AST No AST	32 196	72 (66-78) 70 (62-77)	Diagnosis of NMIBC	3.6	Bladder tumor recurrence and survival
Shiota, 2015, Japan (32)	Retrospective cohort	Chart review	2000-2012	1334	ADT No ADT: RT Surgery	266 631 437	74 (69-78) 70 (65-74) 65 (60-69)	Diagnosis of prostate cancer	3.8	Incidence of bladder cancer
Van Hemelrijck, 2014,Switzerland (33)	Retrospective cohort	Trial database	1980-2010	20559	PCa with SPT PCa without SPT	1718 18841	71.4 ± 7.7 71.7 ± 9.3	Diagnosis of prostate cancer	5	Incidence of bladder cancer
Wallner, 2013, America (34)	Retrospective cohort	Administrative database	1998-2007	24038	PCa with SPT PCa without SPT	1359 22679	60-80 60-80	Diagnosed of localized prostate cancer	5.5	Incidence of bladder cancer
Wang, 2020, Taiwan (35)	Retrospective cohort	Administrative database	1998-2010	5214	5-ARIs No 5-ARIs	474 4740	76.5 ± 7.9 76.6 ± 8.5	Diagnosis of bladder cancer	3	Bladder cancer mortality and recurrence
Wissing, 2021, Canada (36)	Retrospective cohort	Administrative database	2000-2015	2822	5-ARIs No 5-ARIs	284 2538	74 (70-79) 70 (64-76)	Diagnosis of bladder cancer	7.7	Bladder tumor recurrence and survival
Wu, 2019, America (37)	Retrospective cohort	Chart review	2001-2017	274	AST No AST	36 238	68.3 68.3	NMIBC	3.1	Bladder tumor recurrence and survival
Zhu, 2021, America (38)	Retrospective cohort	Administrative database	2000-2016	42406	5-ARIs No 5-ARIs	5698 36708	70 ± 10.9 66.3 ± 13	Diagnosis of BPH	6.1	Incidence of bladder cancer

5-ARIs, 5-alpha reductase inhibitors; AST, Androgen suppression therapy; ADT, Androgen deprivation therapy; NMIBC, Non-muscle-invasive bladder cancer; BCG, Bacille Calmette-Guerin; NA, Not available; LUTS, Lower urinary tract symptoms; TURB, Transurethral resection of bladder; OS, Overall survival; RFS, Recurrence-free survival; CSS, Cancer-specific survival; LVI, Lymphovascular invasion; PNI, Perineural invasion; MTOPS, Medical Treatment of Prostate Symptoms; RT, Radiotherapy; SPT, Second primary tumor; BPH, Benign prostatic hyperplasia; PLCO, Prostate, Lung, Colon, Ovarian.

### Effect of AST on Bladder Cancer Incidence

**Figure 2:**
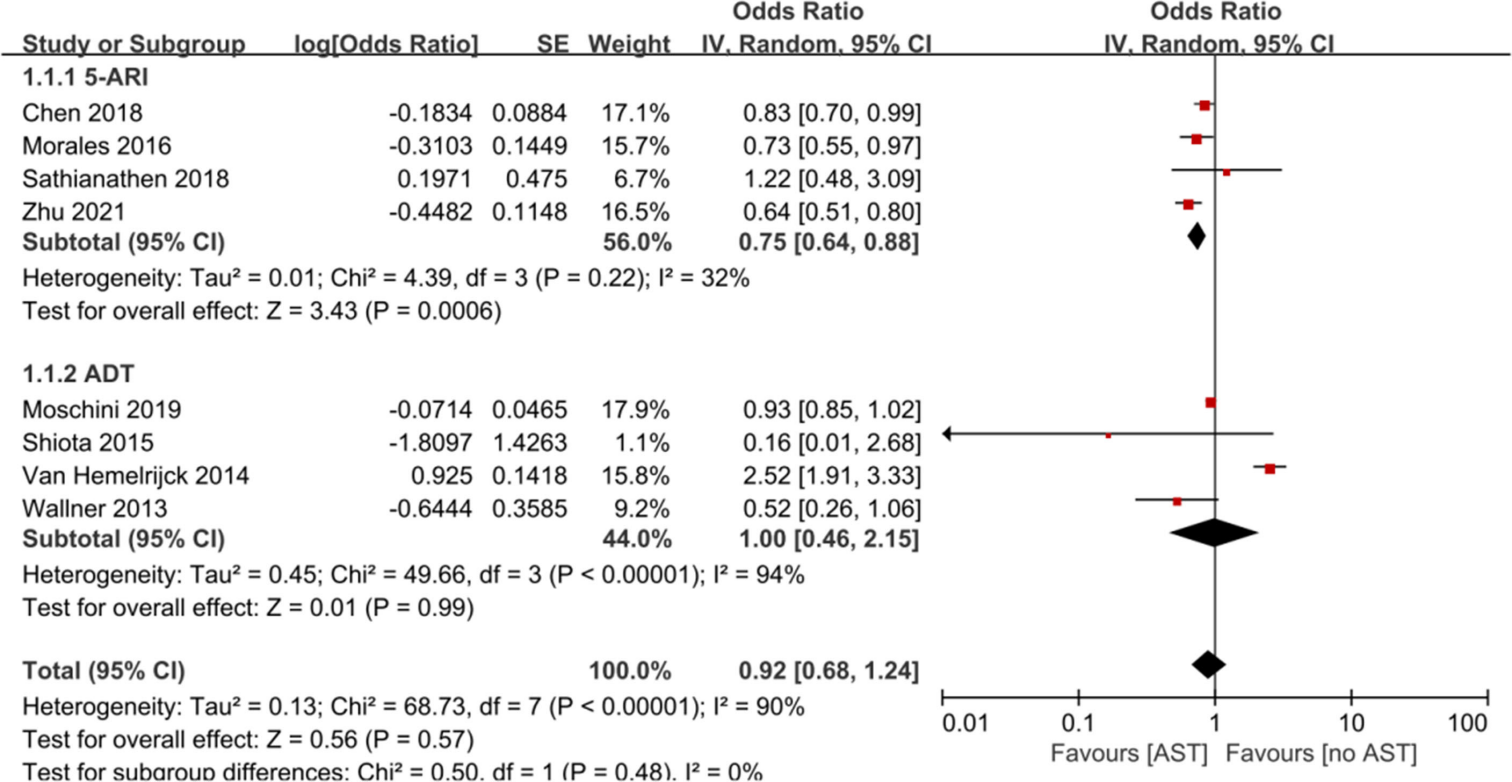
Forest plots showing the effect of AST on bladder cancer incidence. AST, Androgen suppression therapy; 5-ARI, 5-alpha reductase inhibitor; ADT, Androgen deprivation therapy.

Eight studies with 393 907 participants evaluated whether AST reduced the incidence of bladder cancer diagnosis. The results from these studies are summarized in **Table 2**. Three studies reported a protective effect of AST on bladder cancer incidence, four reported no association, and one reported an increased risk. The meta-analysis of studies revealed a nonsignificant reduction in BCa incidence (OR: 0.92, 95% CI: 0.68–1.24) (**Figure 2**). Evidence of statistically significant heterogeneity was found in selected studies (*I^2^* = 90%, p < 0.001). When stratified by the type of AST, we found a statistically lower incidence of bladder cancer among men with finasteride (OR: 0.75, 95% CI: 0.64–0.88), while no statistically significant effect was seen with ADT (OR: 1.00, 95% CI: 0.46–2.15) vs. nonusers. In particular, Chen et al. (22) showed that only patients who received finasteride > 6 months had a lower risk of BCa. In the study of Morales et al. (27), the risk reduction was only observed in well-differentiated and moderately differentiated tumors, while the diagnosis of poorly differentiated or undifferentiated tumors was not reduced. Zhu et al. (38) indicated that the use of finasteride was associated with significant reductions in the risk of high-grade BCa and non-muscle invasive BCa. In addition, a decrease in the risk of BCa was shown only in Caucasians and Hispanics but not among African Americans.

**Table 2:** The effect of androgen suppression therapy on bladder cancer incidence.

Study, year, country	AST	AST duration	Bladder cancer cases (n)	Risk estimate for bladder cancer diagnosis	Notes
Chen, 2018, Taiwan (22)	Finasteride	< 6 months > 6 months	16784	1-179 cDDD OR 0.93 (95% CI: 0.79-1.09). ≥180 cDDD OR 0.84 (95% CI: 0.70-0.99)*	Adjusted for comorbidities (diabetes mellitus, cerebrovascular disease, chronic kidney disease, hypertension and hyperlipidemia), socioeconomic status (low, moderate and high), geographic region (northern, central, southern and eastern)
Morales, 2016, America (27)	Finasteride	>12 months	1031	HR 0.733 (95% CI: 0.552-0.974)*	Adjusted for age, smoking status, body mass index at baseline, race, family history of BCa, randomization arm, colon comorbidity, prostatitis, duration smoked cigarettes, and education
Moschini, 2019, America (28)	ADT	59 months	2495	HR 0.93 (95% CI: 0.85-1.02)	Adjusted for age, race, PCa clinical tumor stage, PCa biopsy Gleason score, as well as marital, socio-economic status and ever-smoker status, and competing-risk mortality
Sathianathen, 2018, America (30)	Finasteride	72 months	18	0.74% with Finasteride vs. 0.61% with control. OR 1.22 (95% CI: 0.48-3.09)	No adjustment of variables due to few events
Shiota, 2015, Japan (32)	ADT	45.5 months	19	0 with ADT vs.1.1% with surgery. OR 0.15 (95% CI: 0.01-2.68)	No adjustment of variables due to few events
Van Hemelrijck, 2014,Switzerland (33)	ADT	60 months	197	SIR 2.54 (95% CI: 1.91-3.33)*	The SIR is defined as the ratio of the observed numbers of primary tumors to the expected numbers
Wallner, 2013, America (34)	GnRH agonist	66 months	132	HR 0.53 (95% CI: 0.26-1.06)	Adjusted for age, race, year of prostate cancer diagnosis, healthcare visits, stage, Gleason score, and radiation therapy
Zhu, 2021, America (38)	Finasteride	73.6 months	846	HR 0.64 (95% CI: 0.51-0.80)*	Adjustment for age, race/ethnicity (Caucasian, African American, Hispanic and other) as well as smoking history

ADT, Androgen deprivation therapy; AST, Androgen suppression therapy; GnRH, Gonadotropin-releasing hormone; BCa, Bladder cancer; cDDD, Cumulative defined daily dose; CI, Confidence interval; SIR, Standardized incidence ratio; HR, Hazard ratio; OR, Odds ratio; PCa, Prostate cancer. *p < 0.05.

### Effect of AST on Bladder Cancer Recurrence and Progression

**Figure 3:**
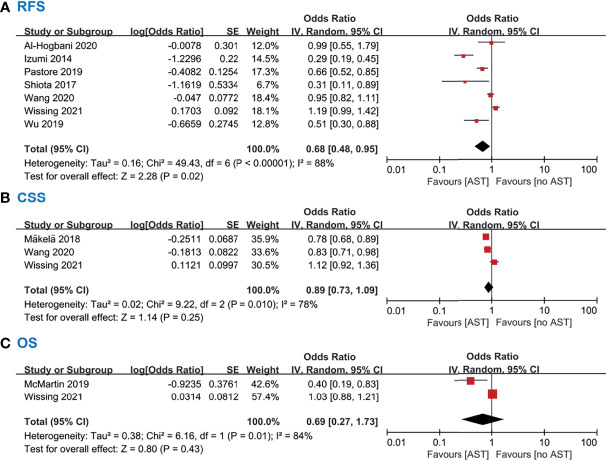
**(A)** Forest plots showing the effect of AST on RFS in bladder cancer. **(B)** Forest plots showing the effect of AST on CSS in bladder cancer. **(C)** Forest plots showing the effect of AST on OS in bladder cancer. AST, Androgen suppression therapy; RFS, Recurrence-free survival; CSS, Cancer-specific survival; OS, Overall survival.

Ten studies including 20 100 participants reported the impact of AST on patients diagnosed with bladder cancer (**Table 3**). Five studies evaluated patients with non-muscle-invasive bladder cancer (NMIBC), and five included all patients with bladder cancer. The manipulation of the androgen signaling pathway involved the use of 5-ARIs in 4263 patients and ADT in 233 patients. For the analysis of RFS, a meta-analysis of seven studies with corresponding HRs was conducted. Compared with nonusers, AST users had significantly improved RFS (HR: 0.68, 95% CI: 0.48–0.95) (**Figure 3A**). The pooled analysis for RFS detected significant heterogeneity (*I^2^* = 90%, *p* < 0.001). Similarly, Kufukihara et al. (24) revealed that the rate of bladder tumor recurrence was significantly lower in the ADT group than in the counterpart (*p* = 0.027). However, McMartin et al. (26) failed to find a significant difference in RFS between patients undergoing therapy with 5-ARIs and controls.

**Table 3:** The effect of androgen suppression therapy on bladder cancer recurrence and progression.

Study, year, country	AST	AST duration	Outcome	Risk estimate	Adjusted for covariates
Al-Hogbani, 2020, Canada (21)	Finasteride or Dutasteride	> 6 months	RFS PFS	HR 1.00 (95% CI: 0.55-1.79) 5-yr PFS with vs. without AST: 97.4% vs 98.2%	Adjusted for age, stage, grade, number of tumors, smoking history, tumor size, presence of CIS, and intravesical treatment
Izumi, 2014, Japan (23)	ADT	62 months	RFS	HR 0.29 (95% CI: 0.19-0.45)*	Adjusted for age. stage, grade, tumor number, tumor size, presence of CIS, and intravesical treatment
Kufukihara, 2021, Japan (24)	ADT	60 months	RFS PFS	5-yr RFS with vs. without ADT: 43.7% vs 27.7% (p = 0.027)* 5-yr PFS with vs. without ADT: p = 0.52	No adjustment of variables due to few events
Mäkelä, 2018, Finland (25)	Finasteride or Dutasteride	24 months	CSS Multiple TURB	Pre-diagnostic 5-ARI use: HR 0.85 (95% CI:0.74-0.97)* Post-diagnostic 5-ARI use: HR 0.78 (95% CI:0.68-0.89)* ≥2 resections: OR 0.89 (95% CI:0.74-1.07) ≥ 5 resections: OR 0.82 (95% CI:0.58-1.16)	Adjusted for age, gender, co-morbidities, primary bladder cancer treatment (surgery vs. other) and tumor extent at diagnosis (localized vs metastatic)
McMartin, 2019, Canada (26)	Finasteride or Dutasteride	22.1 months	OS RFS; CSS LVI; PM; PNI	HR: 0.40 (95% CI: 0.19-0.83)* No significant difference; No significant difference OR: 0.49 (95% CI: 0.2-1.00)*; NS; NS	Adjusted for age, use of neoadjuvant chemotherapy and pathologic stage
Pastore, 2019, Italy (29)	Dutasteride	>12 months	RFS	HR: 0.67 (95% CI: 0.52-0.85)*	Adjusted for age, stage, grade, number of tumors, smoking history, presence of CIS, and intravesical treatment
Shiota, 2017, Japan (31)	GnRH-agonist or Bicalutamide or Dutasteride	28 months	RFS PFS	HR: 0.36 (95% CI: 0.11-0.89)* PFS with vs. without AST: 100% vs 96.9%	Adjusted for stage, number of tumors, size of tumor, smoking status, and intravesical therapy
Wang, 2020, Taiwan (35)	5-ARIs	≥1 months	CSS RFS	OR 0.835 (95% CI: 0.71–0.98)* OR 0.956 (95% CI: 0.82–1.11)	Adjusted for age, and comorbidities including diabetes mellitus, hypertension, chronic kidney disease and hyperlipidemia
Wissing, 2021, Canada (36)	Finasteride or Dutasteride	24 months	OS CSS RFS	HR 1.03 (95% CI: 0.88–1.21) HR 1.12 (95% CI: 0.92–1.36) HR 1.19 (95% CI: 0.99–1.42)	Adjusted for age, region of residence, Charlson’s comorbidity index, year of surgery, driving distance to the hospital, hospital type, annual radical cystectomy volume of the hospital and lead surgeon, type of bladder diversion, and administration of neoadjuvant chemotherapy
Wu, 2019, America (37)	GnRH-agonist or Anti-androgen or 5-ARIs	20 months	RFS PFS	HR: 0.53 (95% CI: 0.30–0.88)* 5-yr PFS with vs without AST: 80% vs 63% (p = 0.23)	Smoking history, risk group (low/intermediate or high), and postoperative chemotherapy use

5-ARIs, 5-alpha reductase inhibitors; ADT, Androgen deprivation therapy; AST, Androgen suppression therapy; GnRH, Gonadotropin-releasing hormone; BCG, Bacille Calmette-Guerin; PFS, Progression-free survival; CIS, Carcinoma in situ; TURB, Transurethral resection of bladder; OS, Overall survival; RFS, Recurrence-free survival; CSS, Cancer-specific survival; LVI, Lymphovascular invasion; PM, Positive margins; PNI, Perineural invasion; CI, Confidence interval; OR, Odds ratio; HR, Hazard ratio; NS, No significance. *p < 0.05.

For the analysis of CSS, a meta-analysis of three studies was conducted. Pooled data for CSS confirmed a nonsignificant difference in patients undergoing therapy with 5-ARIs compared to controls (HR: 0.89, 95% CI: 0.73–1.09) (**Figure 3B**). The pooled analysis found significant heterogeneity (*I^2^* = 78%, *p* = 0.01). Similarly, in the study by McMartin et al. (26), there was no significant difference in CSS between patients undergoing therapy with 5-ARIs and controls. Two studies reported OS in patients with 5-ARIs treatment after diagnosing BCa; there was no significant reduction in OS in these patients (HR: 0.69, 95% CI: 0.27–1.73) (**Figure 3C**). High heterogeneity for OS was observed (*I^2^* = 84%, *p* = 0.01). Owing to a paucity of HR data from PFS, we performed a descriptive analysis. PFS was investigated in 4 studies, and no differences between AST users and nonusers were found (21, 24, 31, 37).

## Discussion

In this comprehensive meta-analysis, we did not find evidence to support the previous hypothesis that the AST is associated with a lower incidence of bladder cancer. Interestingly, subgroup analysis in patients receiving finasteride showed a decreased risk of BCa incidence (OR: 0.75, 95% CI: 0.64–0.88), while ADT had no effect on reducing BCa incidence. In addition, AST significantly reduced RFS in patients with bladder cancer but had no significant effect on CSS, OS or PFS.

Contrary to results of a previous meta-analysis (16), the clinical evidence in this review shows that there is no significant difference in the incidence of BCa between patients with AST and patients without AST. The earlier systematic review and meta-analysis included only three retrospective cohort studies to evaluate the impact of AST on bladder cancer incidence. Obviously, earlier conclusions are easily influenced by the results of newly published studies. In the subgroup analysis based on the type of AST, 5-ARIs exposure was significantly correlated with a decreased risk of subsequent BCa; however, ADT was not significantly correlated with the risk of subsequent BCa. Thus, it seems that suppressing the AR axis more effectively will not yield greater benefits. Nonetheless, we believe that more studies are needed to better evaluate the true benefits of ADT, as this treatment may only affect patients whose BCa does express AR (28). Notably, only a study from Van Hemelrijck et al. (33) indicated an increased risk of bladder cancer associated with ADT use. The authors calculated standardized incidence rates comparing the incidence of second primary malignancies (including bladder cancer) in the prostate cancer (PCa) patient group vs. the general male population in Zurich. However, compared with the general male population, patients with PCa may be monitored more carefully and contact doctors more frequently, thereby increasing the detection rate of bladder cancer and leading to detection bias (39). In our review, bicalutamide was not fully investigated due to limited use of included studies.

The pooled data for RFS in this review show that the risk of BCa recurrence is significantly reduced in patients undergoing hormonal manipulation with AST, which is consistent with the results of previous meta-analyses (15, 16). Creta et al. (15) and Kourbanhoussen et al. (2) indicated that low-grade and low-risk NMIBC may benefit more from the use of AST. This benefit was well reflected in studies including only NMIBC or studies with a high proportion of NMIBC and low-grade patients (23, 29, 31, 37). However, the pooled data for CSS, OS and PFS do not support a protective role of ADT and/or 5-ARIs in terms of BCa progression and survival in the subjects evaluated. Interestingly, Wu et al. (37) indicated that therapy with ADT or 5-ARIs may be associated with lower progression rates in patients with low/intermediate-risk BCa but not in their high-risk counterparts. The negative finding for PFS may be due to the small sample size and the number of events during follow-up, rather than a true lack of correlation. Moreover, the included studies contained many high-risk NMIBC and MIBC patients. It has been reported that these tumor types can reduce dependence on the AR signaling pathway, which may partly explain the lack of association between AST and BCa progression (37, 40–42). As expected, given the prevalence of urinary symptoms in older men, most studies have investigated the role of 5-ARIs in BCa, and only a few studies have investigated the role of ADT in BCa. Because the number of individual studies is insufficient for comparison, it is not clear whether more effective inhibition of the AR axis will yield greater benefits.

Zhu et al. (38) first demonstrated that after using finasteride, although Hispanic men have a similar reduced risk of bladder cancer compared with Caucasian men, African-American men do not. A possible biological explanation for this observation might be the structural variations in the AR protein across different races. African Americans are more likely to contain polymorphisms in AR, which causes their AR to become active and independent of DHT binding (38). The role of AST is not limited to the androgen axis. Clinically, 5-ARIs can increase serum estrogen levels (43, 44). With more effective anti-androgens, the reflex of estrogen increases even higher (2, 45, 46). Estrogens play important roles in BCa development and progression by exerting both stimulatory and inhibitory actions *via* estrogen receptor α (ERα) and ERβ (2, 8). Overall, it appears that estrogens may protect against or inhibit BCa development, but later—at more advanced stages—they might support tumor progression (8). The estrogen-signaling pathway during AST may still partially explain the observed effects in AST.

The number of included studies for meta-analysis was too small to fully assess the publication bias of the effects of AST on incidence or recurrence. Some of heterogeneities were too high. We speculate that several observational studies included in the present meta-analysis did not adjust for potential confounders such as age, race/ethnicity and smoking history, which may bias the pooled effect estimate and may affect heterogeneity. Moreover, differences in AST type, AST exposure time, follow-up duration and demographic characteristics of the included studies are also important reasons for the heterogeneity of results. The proportion of participants receiving 5-ARIs in the currently included studies is obviously higher than that of participants receiving ADT, making the overall effect of outcomes of AST more inclined to 5-ARIs. In most of the included studies, the average age of participants in the AST group was slightly higher than that in the non-AST group. The AST exposure time and follow-up duration of the included literature vary, although it was mostly longer than 2 years. Compared with a duration of less than 6 months, it seems that the use of 5ARIs for greater than 6 months can lead to more significant benefits (22), but the benefits have not been accumulated with years of 5-ARIs use (25). Most of the original studies did not mention the detailed characteristics of BCa, including grade, stage, and cancer cell type, which further limited the pooled analysis. We still do not know very clearly which types of BCa have a lower incidence and which types of BCa have a low recurrence rate after AST. Although the available preclinical evidence demonstrates that AST can interfere with the sensitivity of BCa to BCG or other therapies, its benefit is not observable when given in clinical studies (2, 15, 21). Further research is needed to better evaluate the role of androgen suppression in specific subgroups of BCa patients, to compare the effects of 5-ARIs and ADT and to better clarify androgen manipulation strategies for patients with BCa undergoing BCG, radiation or chemotherapy.

## Conclusion

Our systematic review and meta-analysis identified 18 studies that evaluated androgen suppression on clinical outcomes in BCa patients. AST was not associated with a lower risk of BCa incidence, but a subgroup analysis showed that patients receiving 5-ARIs had a reduced risk of BCa incidence. In addition, AST has a beneficial effect on the recurrence rates of bladder cancer. We did not observe any significant differences in AST on CSS, OS or PFS when compared with the control. Further well-designed prospective studies adjusted for the major and common confounding factors are needed to validate our findings.

## Data Availability Statement

The raw data supporting the conclusions of this article will be made available by the authors, without undue reservation.

## Author Contributions

Conceptualization: PX, HP, and ZD. Data curation: YH, DG, DL, and PX. Formal analysis: WY, MW, PX, ZD, and YL. Project administration: HP. Resources: HP and ZD. Software: PX and YH. Supervision: HP. Validation: PX and HP. Visualization: PX. Writing - original draft: PX. Writing - review & editing: all authors. All authors contributed to the article and approved the submitted version.

## Funding

This work was supported by the National Natural Science Foundation of China (Grant No. 81772698 and 82072833 to HP) and the Open Research Fund from Beijing Advanced Innovation Center for Big Data-Based Precision Medicine, Beijing Tongren Hospital, Beihang University & Capital Medical University (Grant No. BHTR-KFJJ-202005 to HP).

## Conflict of Interest

The authors declare that the research was conducted in the absence of any commercial or financial relationships that could be construed as a potential conflict of interest.

## Publisher’s Note

All claims expressed in this article are solely those of the authors and do not necessarily represent those of their affiliated organizations, or those of the publisher, the editors and the reviewers. Any product that may be evaluated in this article, or claim that may be made by its manufacturer, is not guaranteed or endorsed by the publisher.

## References

[1] Lenis AT Lec PM Chamie K Mshs MD . Bladder Cancer: A Review. JAMA (2020) 324(19):1980–91. doi: 10.1001/jama.2020.17598 33201207

[2] Kourbanhoussen K McMartin C Lodde M Zlotta A Bryan RT Toren P . Switching Cancers: A Systematic Review Assessing the Role of Androgen Suppressive Therapy in Bladder Cancer. Eur Urol Focus (2020) 7(5):1044–51. doi: 10.1016/j.euf.2020.10.002 33132108

[3] Dobruch J Daneshmand S Fisch M Lotan Y Noon AP Resnick MJ . Gender and Bladder Cancer: A Collaborative Review of Etiology, Biology, and Outcomes. Eur Urol (2016) 69(2):300–10. doi: 10.1016/j.eururo.2015.08.037 26346676

[4] Ide H Miyamoto H . Sex Hormone Receptor Signaling in Bladder Cancer: A Potential Target for Enhancing the Efficacy of Conventional Non-Surgical Therapy. Cells (2021) 10(5):1169. doi: 10.3390/cells10051169 34064926 PMC8150801

[5] Mori K Mostafaei H Enikeev DV Lysenko I Quhal F Kimura S . Differential Effect of Sex on Outcomes After Radical Surgery for Upper Tract and Bladder Urothelial Carcinoma: A Systematic Review and Meta-Analysis. J Urol (2020) 204(1):58–62. doi: 10.1097/JU.0000000000000788 31995432

[6] Uhlig A Strauss A Seif Amir Hosseini A Lotz J Trojan L Schmid M . Gender-Specific Differences in Recurrence of Non-Muscle-Invasive Bladder Cancer: A Systematic Review and Meta-Analysis. Eur Urol Focus (2018) 4(6):924–36. doi: 10.1016/j.euf.2017.08.007 28888813

[7] Martinez-Rojo E Berumen LC Garcia-Alcocer G Escobar-Cabrera J . The Role of Androgens and Androgen Receptor in Human Bladder Cancer. Biomolecules (2021) 11(4):594. doi: 10.3390/biom11040594 33919565 PMC8072960

[8] Costa AR Lanca de Oliveira M Cruz I Goncalves I Cascalheira JF Santos CRA . The Sex Bias of Cancer. Trends Endocrinol Metab (2020) 31(10):785–99. doi: 10.1016/j.tem.2020.07.002 32900596

[9] Moorthy HK Prabhu GGL Venugopal P . Clinical and Therapeutic Implications of Sex Steroid Hormone Receptor Status in Urothelial Bladder Cancer. Indian J Urol (2020) 36(3):171–8. doi: 10.4103/iju.IJU_320_19 PMC7531383 33082631

[10] Koti M Ingersoll MA Gupta S Lam CM Li X Kamat AM . Sex Differences in Bladder Cancer Immunobiology and Outcomes: A Collaborative Review With Implications for Treatment. Eur Urol Oncol (2020) 3(5):622–30. doi: 10.1016/j.euo.2020.08.013 32967818

[11] Luna-Velez MV Dijkstra JJ Heuschkel MA Smit FP van de Zande G Smeets D . Androgen Receptor Signalling Confers Clonogenic and Migratory Advantages in Urothelial Cell Carcinoma of the Bladder. Mol Oncol (2021) 15(7):1882–900. doi: 10.1002/1878-0261.12957 PMC8253097 33797847

[12] Deng G Wang R Sun Y Huang CP Yeh S You B . Targeting Androgen Receptor (AR) With Antiandrogen Enzalutamide Increases Prostate Cancer Cell Invasion Yet Decreases Bladder Cancer Cell Invasion *via* Differentially Altering the AR/CircRNA-ARC1/MiR-125b-2-3p or Mir-4736/Ppargamma/MMP-9 Signals. Cell Death Differ (2021) 28(7):2145–59. doi: 10.1038/s41418-021-00743-w PMC8257744 34127806

[13] Ide H Inoue S Mizushima T Jiang G Chuang KH Oya M . Androgen Receptor Signaling Reduces Radiosensitivity in Bladder Cancer. Mol Cancer Ther (2018) 17(7):1566–74. doi: 10.1158/1535-7163.Mct-17-1061 29720561

[14] Tripathi A Gupta S . Androgen Receptor in Bladder Cancer: A Promising Therapeutic Target. Asian J Urol (2020) 7(3):284–90. doi: 10.1016/j.ajur.2020.05.011 PMC7385521 32742928

[15] Creta M Celentano G Napolitano L La Rocca R Capece M Califano G . Inhibition of Androgen Signalling Improves the Outcomes of Therapies for Bladder Cancer: Results From a Systematic Review of Preclinical and Clinical Evidence and Meta-Analysis of Clinical Studies. Diagn (Basel) (2021) 11(2):351. doi: 10.3390/diagnostics11020351 PMC7923424 33672461

[16] Kim A Kim MS Ahn JH Choi WS Park HK Kim HG . Clinical Significance of 5-Alpha Reductase Inhibitor and Androgen Deprivation Therapy in Bladder Cancer Incidence, Recurrence, and Survival: A Meta-Analysis and Systemic Review. Aging Male (2020) 23(5):971–8. doi: 10.1080/13685538.2019.1646238 31724468

[17] Tellini R Mari A Muto G Cacciamani GE Ferro M Stangl-Kremser J . Impact of Smoking Habit on Perioperative Morbidity in Patients Treated With Radical Cystectomy for Urothelial Bladder Cancer: A Systematic Review and Meta-Analysis. Eur Urol Oncol (2020) 4(4):580–93. doi: 10.1016/j.euo.2020.10.006 33160975

[18] Ronksley PE Brien SE Turner BJ Mukamal KJ Ghali WA . Association of Alcohol Consumption With Selected Cardiovascular Disease Outcomes: A Systematic Review and Meta-Analysis. BMJ (2011) 342:d671. doi: 10.1136/bmj.d671 21343207 PMC3043109

[19] Siristatidis C Sergentanis TN Kanavidis P Trivella M Sotiraki M Mavromatis I . Controlled Ovarian Hyperstimulation for IVF: Impact on Ovarian, Endometrial and Cervical Cancer–a Systematic Review and Meta-Analysis. Hum Reprod Update (2013) 19(2):105–23. doi: 10.1093/humupd/dms051 23255514

[20] Zhang J Yu KF . What’s the Relative Risk? A Method of Correcting the Odds Ratio in Cohort Studies of Common Outcomes. JAMA (1998) 280(19):1690–1. doi: 10.1001/jama.280.19.1690 9832001

[21] Al-Hogbani M Gilbert S Lodde M Fradet Y Toren P . Does 5-Alpha Reductase Inhibitor Use Improve the Efficacy of Intravesical Bacille Calmette-Guérin (BCG) for Non-Muscle Invasive Bladder Cancer? Bladder Cancer (2020) 6(1):63–9. doi: 10.3233/blc-190262

[22] Chen CC Huang CP Tsai YT Hseih TF Shyr CR . The Genomic Alterations of 5alpha-Reductases and Their Inhibitor Finasteride’s Effect in Bladder Cancer. Anticancer Res (2017) 37(12):6893–8. doi: 10.21873/anticanres.12152 29187470

[23] Izumi K Taguri M Miyamoto H Hara Y Kishida T Chiba K . Androgen Deprivation Therapy Prevents Bladder Cancer Recurrence. Oncotarget (2014) 5(24):12665–74. doi: 10.18632/oncotarget.2851 PMC4350350 25557268

[24] Kufukihara R Kikuchi E Ogihara K Shigeta K Yanai Y Takamatsu K . Role of Previous Malignancy History in Clinical Outcomes in Patients With Initially Diagnosed Non-Muscle Invasive Bladder Cancer. Ann Surg Oncol (2021) 28(9):5349–59. doi: 10.1245/s10434-021-09750-0 33666810

[25] Makela VJ Kotsar A Tammela TLJ Murtola TJ . Bladder Cancer Survival of Men Receiving 5alpha-Reductase Inhibitors. J Urol (2018) 200(4):743–8. doi: 10.1016/j.juro.2018.04.082 29730200

[26] McMartin C Lacombe L Fradet V Fradet Y Lodde M Toren P . Receipt of 5-Alpha Reductase Inhibitors Before Radical Cystectomy: Do They Render High-Grade Bladder Tumors Less Aggressive? Clin Genitourin Cancer (2019) 17(6):e1122–8. doi: 10.1016/j.clgc.2019.07.016 31594737

[27] Morales EE Grill S Svatek RS Kaushik D Thompson IM Jr. Ankerst DP . Finasteride Reduces Risk of Bladder Cancer in a Large Prospective Screening Study. Eur Urol (2016) 69(3):407–10. doi: 10.1016/j.eururo.2015.08.029 26320383

[28] Moschini M Zaffuto E Karakiewicz P Mattei A Gandaglia G Fossati N . The Effect of Androgen Deprivation Treatment on Subsequent Risk of Bladder Cancer Diagnosis in Male Patients Treated for Prostate Cancer. World J Urol (2019) 37(6):1127–35. doi: 10.1007/s00345-018-2504-3 30276543

[29] Pastore AL Fuschi A De Nunzio C Balzarro M Al Salhi Y Velotti G . Possible Role of 5-Alpha Reductase Inhibitors in Non-Invasive Bladder Urothelial Neoplasm: Multicentre Study. Minerva Urol Nefrol (2019). doi: 10.23736/S0393-2249.19.03563-X 31833718

[30] Sathianathen NJ Fan Y Jarosek SL Lawrentschuk NL Konety BR . Finasteride Does Not Prevent Bladder Cancer: A Secondary Analysis of the Medical Therapy for Prostatic Symptoms Study. Urol Oncol (2018) 36(7):338.e13–338.e17. doi: 10.1016/j.urolonc.2018.03.020 29731413

[31] Shiota M Kiyoshima K Yokomizo A Takeuchi A Kashiwagi E Dejima T . Suppressed Recurrent Bladder Cancer After Androgen Suppression With Androgen Deprivation Therapy or 5alpha-Reductase Inhibitor. J Urol (2017) 197(2):308–13. doi: 10.1016/j.juro.2016.08.006 27506696

[32] Shiota M Yokomizo A Takeuchi A Imada K Kiyoshima K Inokuchi J . Secondary Bladder Cancer After Anticancer Therapy for Prostate Cancer: Reduced Comorbidity After Androgen-Deprivation Therapy. Oncotarget (2015) 6(16):14710–9. doi: 10.18632/oncotarget.3817 PMC4546499 25900243

[33] Van Hemelrijck M Feller A Garmo H Valeri F Korol D Dehler S . Incidence of Second Malignancies for Prostate Cancer. PLoS One (2014) 9(7):e102596. doi: 10.1371/journal.pone.0102596 25047238 PMC4105414

[34] Wallner LP Wang R Jacobsen SJ Haque R . Androgen Deprivation Therapy for Treatment of Localized Prostate Cancer and Risk of Second Primary Malignancies. Cancer Epidemiol Biomarkers Prev (2013) 22(2):313–6. doi: 10.1158/1055-9965.EPI-12-1137 PMC3758254 23292083

[35] Wang CS Li CC Juan YS Wu WJ Lee HY . 5alpha-Reductase Inhibitors Impact Prognosis of Urothelial Carcinoma. BMC Cancer (2020) 20(1):872. doi: 10.1186/s12885-020-07373-4 32917158 PMC7488389

[36] Wissing MD O’Flaherty A Dragomir A Tanguay S Kassouf W Aprikian AG . The Use of 5-Alpha Reductase Inhibitors and Alpha-1 Blockers Does Not Improve Clinical Outcome in Male Patients Undergoing Radical Cystectomy for Bladder Cancer in Quebec, Canada. Clin Genitourin Cancer (2021) 19(4):371–371.e9. doi: 10.1016/j.clgc.2021.01.007 33676834

[37] Wu SC Kwon D Jue JS Chen FV Velasquez Escobar MC Punnen S . Androgen Suppression Therapy Is Associated With Lower Recurrence of Non-Muscle-Invasive Bladder Cancer. Eur Urol Focus (2021) 7(1):142–7. doi: 10.1016/j.euf.2019.04.021 31103602

[38] Zhu D Srivastava A Agalliu I Fram E Kovac EZ Aboumohamed A . Finasteride Use and Risk of Bladder Cancer in a Multiethnic Population. J Urol (2021) 206(1):15–21. doi: 10.1097/JU.0000000000001694 33617325

[39] Santella C Rouette J Brundage MD Filion KB Azoulay L . Androgen Deprivation Therapy for Prostate Cancer and the Risk of Bladder Cancer: A Systematic Review of Observational Studies. Urol Oncol (2020) 38(11):816–25. doi: 10.1016/j.urolonc.2020.04.028 32654949

[40] Miyamoto H Yao JL Chaux A Zheng Y Hsu I Izumi K . Expression of Androgen and Oestrogen Receptors and Its Prognostic Significance in Urothelial Neoplasm of the Urinary Bladder. BJU Int (2012) 109(11):1716–26. doi: 10.1111/j.1464-410X.2011.10706.x 22221549

[41] Sikic D Breyer J Hartmann A Burger M Erben P Denzinger S . High Androgen Receptor mRNA Expression Is Independently Associated With Prolonged Cancer-Specific and Recurrence-Free Survival in Stage T1 Bladder Cancer. Transl Oncol (2017) 10(3):340–5. doi: 10.1016/j.tranon.2017.01.013 PMC5367846 28342317

[42] Boorjian S Ugras S Mongan NP Gudas LJ You X Tickoo SK . Androgen Receptor Expression Is Inversely Correlated With Pathologic Tumor Stage in Bladder Cancer. Urology (2004) 64(2):383–8. doi: 10.1016/j.urology.2004.03.025 15302512

[43] Lee YR Im E Kim H Lew BL Sim WY Lee J . Untargeted Metabolomics and Steroid Signatures in Urine of Male Pattern Baldness Patients After Finasteride Treatment for a Year. Metabolites (2020) 10(4):131. doi: 10.3390/metabo10040131 PMC7241081 32235609

[44] Kristal AR Till C Tangen CM Goodman PJ Neuhouser ML Stanczyk FZ . Associations of Serum Sex Steroid Hormone and 5alpha-Androstane-3alpha,17beta-Diol Glucuronide Concentrations With Prostate Cancer Risk Among Men Treated With Finasteride. Cancer Epidemiol Biomarkers Prev (2012) 21(10):1823–32. doi: 10.1158/1055-9965.EPI-12-0695 PMC3467348 22879203

[45] Wadhwa VK Weston R Parr NJ . Bicalutamide Monotherapy Preserves Bone Mineral Density, Muscle Strength and has Significant Health-Related Quality of Life Benefits for Osteoporotic Men With Prostate Cancer. BJU Int (2011) 107(12):1923–9. doi: 10.1111/j.1464-410X.2010.09726.x 20950306

[46] Liang Z Cao J Tian L Shen Y Yang X Lin Q . Aromatase-Induced Endogenous Estrogen Promotes Tumour Metastasis Through Estrogen Receptor-Alpha/Matrix Metalloproteinase 12 Axis Activation in Castration-Resistant Prostate Cancer. Cancer Lett (2019) 467:72–84. doi: 10.1016/j.canlet.2019.09.001 31499120

