# Supplementary material for: Impact of Androgen Suppression Therapy on the Risk and Prognosis of Bladder Cancer: A Systematic Review and Meta-Analysis
Source: Front Oncol. 2021 Dec 14;11:784627. doi: 10.3389/fonc.2021.784627 (PMC8712679; doi:10.3389/fonc.2021.784627)
Supplement: Supplementary file 1 [file Table_1.docx]

**Supplementary Table 1** Summary of risk of bias among included studies using Risk Of Bias In Non-randomized Studies - of Interventions(ROBINS-I) tool.

| Study, year | Bias due to confounding | Bias in selection of participants into the study | Bias in classification of interventions | Bias due to deviations from intended interventions | Bias due to missing data | Bias in measurement of outcomes | Bias in selection of the reported result | **Overall** |
| --- | --- | --- | --- | --- | --- | --- | --- | --- |
| Al-Hogbani[21], 2020 | Moderate | Low | Moderate | Moderate | Low | Low | Low | Moderate |
| Chen[22], 2018 | Moderate | Moderate | Moderate | Moderate | Moderate | Low | Low | Moderate |
| Izumi[23], 2014 | Moderate | Moderate | Moderate | Low | Low | Moderate | Low | Moderate |
| Kufukihara[24], 2021 | Serious | Serious | Moderate | Moderate | Low | Moderate | Moderate | Serious |
| Mäkelä[25], 2018 | Moderate | Moderate | Low | Moderate | Low | Low | Low | Moderate |
| McMartin[26], 2019 | Moderate | Low | Low | Moderate | Low | Moderate | Moderate | Moderate |
| Morales[27], 2016 | Moderate | Low | Low | Low | Low | Low | Low | Moderate |
| Moschini[28], 2019 | Moderate | Low | Low | Low | Low | Moderate | Low | Moderate |
| Pastore[29], 2019 | Moderate | Low | Moderate | Low | Low | Low | Moderate | Moderate |
| Sathianathen[30], 2018 | Moderate | Low | Low | Low | Moderate | Low | Low | Moderate |
| Shiota[31], 2017 | Moderate | Low | Low | Low | Low | Moderate | Low | Moderate |
| Shiota[32], 2015 | Serious | Serious | Low | Moderate | Low | Moderate | Moderate | Serious |
| Van Hemelrijck[33], 2014 | Serious | Moderate | Low | Moderate | Low | Moderate | Serious | Serious |
| Wallner[34], 2013 | Moderate | Moderate | Low | Low | Low | Moderate | Moderate | Moderate |
| Wang[35], 2020 | Moderate | Moderate | Low | Low | Low | Low | Moderate | Moderate |
| Wissing[36], 2021 | Moderate | Low | Low | Moderate | Low | Low | Moderate | Moderate |
| Wu[37], 2019 | Moderate | Low | Low | Low | Serious | Low | Low | Moderate |
| Zhu[38], 2021 | Moderate | Low | Low | Low | Low | Low | Low | Moderate |
