# Supplementary material for: Impact of Androgen Suppression Therapy on the Risk and Prognosis of Bladder Cancer: A Systematic Review and Meta-Analysis
Source: Front Oncol. 2021 Dec 14;11:784627. doi: 10.3389/fonc.2021.784627 (PMC8712679; doi:10.3389/fonc.2021.784627)
Supplement: Supplementary file 2 [file Table_2.docx]

**Supplementary Table 2** – Search strategy

| PubMed: from inception to June 20, 2021 | | Embase: from inception to June 20, 2021 | |
| --- | --- | --- | --- |
| 1. alpha-reductase inhibitor OR 5α-reductase OR 5ARI OR finasteride OR dutasteride OR androgen deprivation therapy OR androgen suppression therapy OR anti-androgen OR bicalutamide OR enzalutamide OR abiraterone OR GnRH agonist OR GnRH antagonist OR castration OR nilutamide OR flutamide OR apalutamide OR darolutamide) AND (bladder cancer OR urothelial carcinoma OR bladder neoplasms） | **788** | **A:** 5 alpha reductase inhibitor OR '5α reductase' OR 5ARI OR finasteride OR dutasteride OR (androgen AND deprivation AND therapy) OR (androgen AND suppression AND therapy) OR 'anti androgen' OR bicalutamide OR enzalutamide OR abiraterone OR (gnrh AND agonist) OR (gnrh AND antagonist) OR castration OR nilutamide OR flutamide OR apalutamide OR darolutamide | 98193 |
|  |  | **B:** (bladder AND cancer) OR (urothelial AND carcinoma) OR (bladder AND neoplasms) | 133218 |
|  |  | **A** AND **B** | 1842 |
|  |  | **Choose:** “Article” and “Article in press” | **732** |
